# Prey size reflected in tooth wear: a comparison of two wolf populations from Sweden and Alaska

**DOI:** 10.1098/rsfs.2023.0070

**Published:** 2024-06-07

**Authors:** Ellen Schulz-Kornas, Mirella H. Skiba, Thomas M. Kaiser

**Affiliations:** ^1^ Department of Cariology, Endodontology and Periodontology, Liebigstraße 12, University of Leipzig, Leipzig 04103, Germany; ^2^ Department of Vertebrates, Section Mammalogy and Paleoanthropology, Martin-Luther-King-Platz 3, Leibniz Institute for the Analysis of Biodiversity Change (LIB), Hamburg 20146, Germany; ^3^ Institute of Cell and Systems Biology of Animals, Martin-Luther-King-Platz 3, University of Hamburg, Hamburg 20146, Germany

**Keywords:** *Canis lupus*, 3D surface texture, feeding trait, tooth function, antagonistic asymmetry

## Abstract

Ingesta leaves distinct patterns on mammalian teeth during mastication. However, an unresolved challenge is how to include intraspecific variability into dietary reconstruction and the biomechanical aspects of chewing. Two extant populations of the grey wolf (*Canis lupus*), one from Alaska and one from Sweden, were analysed with consideration to intraspecific dietary variability related to prey size depending on geographical origin, sex and individual age as well as tooth function. Occlusal enamel facets of the upper fourth premolars, first molars and the second lower molar were analysed via three-dimensional surface texture analysis. The Swedish wolves displayed facets characterized by higher peaks and deeper, more voluminous dales, featuring an overall rougher surface than the wolves from Alaska. Compared to females, the Swedish male wolves had a slightly larger dale area and hill volume on their facets. Upper fourth premolars are smoother and had higher values in texture direction compared to upper first molars. The upper first molars were rougher than the occluding lower second molars and were characterized by larger and deeper dales. We find evidence supporting intraspecific dietary segregation, and antagonistic asymmetry in occlusal wear signatures. The data offer new insights into the roles of apex predators like the grey wolf.

## 1. Introduction

Carnivora, a highly diverse order within mammals [1], inhabit every climatic zone and range in size from 40 g of the least weasel to 500 kg of a polar bear [2]. With reference to dietary adaptations, most dietary studies on carnivora have used cranial and morphological characters referring to dietary adaptations [3–6], or have performed inter-specific dietary trait reconstruction [7–10]. During the last 15 years, especially, robust three-dimensional approaches to quantifying dental microwear have been applied in Carnivora [7,9]. These studies demonstrate the great potential of dental microwear texture analysis (DMTA) in discriminating between carnivores with different diets, indicating that wear-induced textures may be very promising for detecting interspecific dietary traits.

Within the mammalian order Carnivora (canids, felids, hyenids, mustelids, ursids and pinnipeds), various dietary classification systems are employed to describe carnivory. These systems include different cut-offs, sub-categories with or without invertebrates; for a comprehensive review, see Pollard and Puckett [11]. In this context, the term ‘carnivore’ is used to reflect the percentage of prey species in the diet of *Canis lupus*, as reported by Ballard *et al*. [12], Olsson *et al*. [13] and Müller [14]. Schubert *et al*. [9] recognize the influence of bone consumption on wear textures of carnivores with a well-known diet. This indicates that hyenas, feeding also on the bones of carcasses, are distinct in terms of their wear textures when compared to meat-eaters such as cheetahs. Lions, feeding on both bone and meat, exhibit mixed texture characteristics.

Ungar *et al*. [10] expanded the range of the comparative carnivore dataset by adding coyotes (*Canis latrans*), which display more length-scale anisotropy of relief (*epLsar*, ‘anisotropy’) in their textures, and African wild dogs (*Lycaon pictus*), which possess high levels of heterogeneity of area-scale fractal complexity (*Hasfc*, ‘heterogeneity’). It has been indicated that although DMTA of tooth facets in canids (coyotes and wolves) captures dietary differences [15], Tanis *et al*. [15] also demonstrate that DMTA is indistinguishable between analogous occlusal surfaces (grinding of the m1 talonid to grinding of the m2). There is however a second dental microwear texture approach, which uses three-dimensional industrial areal surface texture standards (3DST, for a review see [16]). This approach is very sensitive, not only at an intraspecific level but also when distinguishing between analogous surfaces of adjoining tooth positions [17–19]. In addition, there is the first evidence that the dietary behaviour of the modern population of the North American grey wolf (*Canis lupus*) has changed over the past 50 years, linked to winter severity and climate change [20]. Thus, our goal is to differentiate dietary adaptations between two modern but geographically distinct populations of the northern hemisphere at an intraspecific level. This will allow us to create a clearer picture of how variable wear textures can be within a species and to explore the dietary habits of local populations to provide a better basis for reconstructing dietary traits in fossil records.

**Figure 1.**
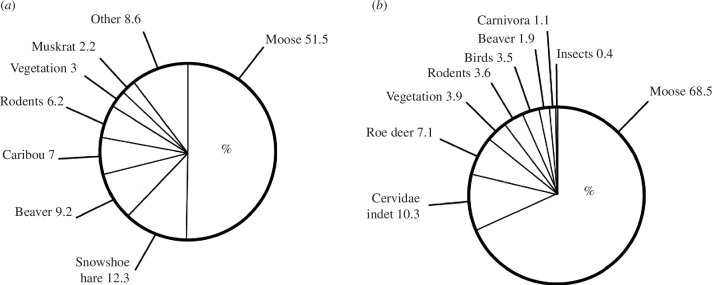
Prey species of *C. lupus* (in %) from (*a*) Alaska (according to Ballard *et al*. [12]), and (*b*) Sweden (according to Olsson *et al*. [13], Müller [14]).

For many apex predators, which play a critical role in maintaining the health of extant ecosystems and food webs, the range of dietary strategies and local traits represented are of great importance. The modern grey wolf (*C. lupus*) was one of the world’s most widely distributed apex predators in the Late Quaternary, with a historical range that covered most of the northern hemisphere [21]. Currently, its distribution has been reduced by about one-third over the last centuries and wolves are found primarily in wilderness and remote areas, most of them in Canada, Alaska and the northern USA, Europe and Asia from about 75°N–12°N [22]. One of the most important questions that remains unanswered is the nature of their interaction with prey populations [23]. Therefore, we aimed to capture a broad range of dietary variation between two geographically distinct populations from Alaska and Sweden to identify the potential for diet-related intraspecific adaptation [22,24,25]. Furthermore, we tested for functional differentiation within a tooth row, to better understand the masticatory process. The diet of wolves from Alaska and Sweden (figure 1) consists mainly of large ungulates such as moose (*Alces alces*) [12–14,26] and other prey species, including roe deer (*Capreolus capreolus*) in Sweden, caribous (*Rangifer tarandus*) in Alaska, beavers (*Castor fiber*), badgers (*Meles meles*), hares (*Lepus europaeus, L. timidus, L. americanus*), small rodents (*Ondatra zibethicus*, *Apodemus* spp., *Microtus spec*., *Sciurus* species, etc.) and diverse birds, as well as parts of vegetation [13,27,28]. In Alaska (figure 1*a*, table 1), their diet consists of 50% large mammals (51.5%, moose) and 50% medium-sized species (7%, caribou) and small mammals (29.9%), vegetation (3%) and others (8.6%) [12], while in Sweden (figure 1*b*, table 1), the main prey is large mammals (68.5%, moose), medium-sized cervids (10.3%) and roe deer (7.1%). Small mammals and birds are consumed to a lesser extent (10.2%) [12–14]. It can be assumed that large carcasses like moose have to be torn to pieces, and meat and softer tissues have to be ripped off [12,29]. Canids use their carnassial teeth (fourth upper premolar and lower first molar) to slice meat and tendons. Bones, however, are being cursed more posteriorly, between the upper first and lower second molars [8,10].

**Table 1.** Body mass of the prey animals from Alaska and Sweden [2] and the mean body mass of all prey, calculated with the data from Olsson *et al*. [13], Ballard *et al*. [12] and Müller [14].

species	body mass (kg)
Alaska	
moose	360
caribou	120
beaver	20
snow hare	5
muskrat	1.5
rodents	1
mean	196.36
Sweden	
moose	360
Cervidae	50
roe deer	30
beaver	20
rodents	1
birds	1
mean	253.30

The feeding sequence of wolves is described as follows: after feeding on the soft internal organs, the large muscles of the hindquarters and forequarters are consumed next, before moving on to head flesh, hind limbs, forelimbs and head content [29]. The tissues of the ribcage, leg bones and hide are consumed last. This sequence is a result of the energetic cost of extracting tissues in relation to the calories gained, and is consistent in all larger carnivores such as hyenas and lions [30]. Regardless of the type of prey, animal food is always contaminated with abrasives.

Dirt and grit particles contaminate fur, feathers and exposed soft tissue. However, the contamination on small prey animals does not come into contact with the teeth as these animals are swallowed whole without chewing [12,29]. To eliminate parasites, wolves also eat plants which make a minor contribution to their diet [12,29]. There is recent evidence for regional and seasonal dietary variation. For example, depending on the season, wolves in southern boreal ecosystems rely more on vegetative matter, e.g. berries, than previously thought [31]. In addition, it was found that wolves could use marine resources as well [32,33] and have substantial regional dietary diversity depending on habitat composition season, vegetation patch interspersion [33].

### 1.1. Aims and hypotheses

#### 1.1.1. Hypothesis 1 (intraspecific dietary segregation)

The individuals from Sweden [13,14] and Alaska [2,12] represent independent wolf populations without frequently migrating animals. We aimed to test if intraspecific variation related to local dietary conditions in geographically separated populations could be documented using three-dimensional surface texture analysis. Even though the diet of beavers and rodents as well as large prey animals, such as moose, roe deer (in Sweden) and caribou (in Alaska), is quite similar in size and build, the proportions of prey groups in the diet differ. In Alaska, 50% of the wolf’s prey is moose and caribou. In Sweden, nearly 70% of the prey is moose, the rest consisting of a large diversity of other orders. The percentage of moose, with their mean body mass of 360 kg, results in an ingesta potentially featuring mechanically more challenging bones, however, observation by Severtsov *et al.* [34] suggests, that the skull, the vertebrate column, the pelvis and the leg bones of elks mostly remained uneaten in the Siberian population they observed.

Considering functional blocks of the wolf dentition as defined by Severtsov *et al.* [34], the premolars form the most homogeneous block of the dental system. Their main function would be holding objects. This function is implemented as the animals pull on parts of the prey, tear pieces off and carry them. The carnassials (P4 and m1) and the anterior molars would disintegrate foods, while the carnassials are used to break bones and to cut through dense connective tissue, such as tendons; moreover, they may also be involved in the disintegration of the food bolus grabbed by the anterior teeth. The talonid of m1 and the posterior molars disintegrate the food further.

Considering the stress-loaded interaction with hard tissues of greater mass and mechanical resistance, we propose frequent tooth–bone and tooth–cartilage contact, in turn leading to occlusal three-dimensional surface textures, also in the posterior molars, showing characteristics like high volume parameters, also found in durophagous taxa. While meat-dominated ingesta, available without engagement in stress-loaded hard tissue interaction produces polished and highly anisotropic (linearly-oriented) wear patterns, a highly durophagous diet, in turn, leaves high volume dales and craters as a result of the impact and crushing action.

We thus expect parameters describing height and volume and indicating high levels of textural roughness would be high in the Swedish population as compared with wolves from Alaska. Because there is only minor sexual dimorphism within the rank of the pack this should also be reflected in the feeding behaviour. We thus expect only little influence of sex ratios and sexual dimorphism on the population-specific signal.

#### 1.1.2. Hypothesis 2 (tooth function)

The teeth of wolves are highly efficient in processing freshly killed prey animals and carcasses [29]. The slicing of meaty parts and softer tissues is performed with a shear-cutting movement using the occluding carnassials. Both teeth (P4 and m1) feature self-sharpening blades when being exposed to ingesta at a high angle, functioning similarly to a pair of scissors [8,35]. Harder materials like bone and cartilage, which have to be crushed to access the nutrient-rich marrow, are puncture crushed between the cusps of the massive first upper molar (M1) and its antagonist the second lower molar (m2, figure 1*a,b*) [8,10,35].

We test the hypothesis, that carnassials shear-cutting action (P4 and m1) and crushing movements (M1 and m2) would lead to distinct DMTA signatures on the dental antagonists involved (hypothesis 2). We expect shear-cutting between the P4 and m1 to be a highly precision-guided function, leading to longitudinal surface texture orientation on the P4 following the orthal chewing stroke. The less guided crushing movements between M1 and m2 are expected to be characterized by multidirectional tooth contacts, contact with hard tissues, as well as traces of ingesta-related particle contacts. This should result in rougher and more voluminous surface textures (e.g. dale structures) on the M1 and a texture direction below 45°. Because of the different functional traits featured by individual tooth positions as well as the precise occlusion of upper and lower teeth, we expected more similarities in textures between the antagonistic M1 and m2 than between the P4 and m1 and propose that the latter would be functionally more distinct.

#### 1.1.3. Hypothesis 3 (antagonistic symmetry)

We further propose the hypothesis that in particular stress-loaded interaction with hard tissues of greater mass and mechanical resistance would differentially affect the symmetry of occlusal wear signatures specifically at cusp positions of M1 and m2 where such contacts occur on a regular basis, provided sufficiently large prey items are being processed (hypothesis 3).

The formation of textures starts with the grasping of and ripping off of prey tissues, followed by ingestion and finally mastication. All those oral activities are performed by orthal jaw movements resulting in a closing stroke that leads to the compression and puncture of tissues and may result in first hard tissue contacts, subsequently providing the grip for pulling and shaking activity during the killing and prey processing phase. Prey tissues are ripped off owing to sharp movements of both the wolf and the prey that tries to escape, and both of these movements contribute to the pulling force. While tissues are compressed between upper and lower cusps, the cranium will introduce the forces of the neck musculature immediately towards upper cusps resulting in lateral movements of those cusps versus ingesta under load, while lower cusps will experience those forces only indirectly, via the jaw joint, limiting peak forces and buffering lateral movements significantly. Lower cusp–ingesta contacts are thus proposed to be always less immediately prone to lateral peak loads as compared to upper cusp–ingesta contacts during the prey processing phase. They should thus rather acts as stable antagonist with much less lateral components of movement, thereby increasing the probability that contacts remain in the same position in relation to the lower dentition while the upper contacts perform a lateral component of friction. We propose this lateral component, which would introduce additional wear to upper cusps to be positively related to prey size, because loads transmitted between teeth and prey are also. Large prey should thus increase differential DMTA signals in antagonistic upper and lower molar cusps.

## 2. Material and methods

**Figure 2.**
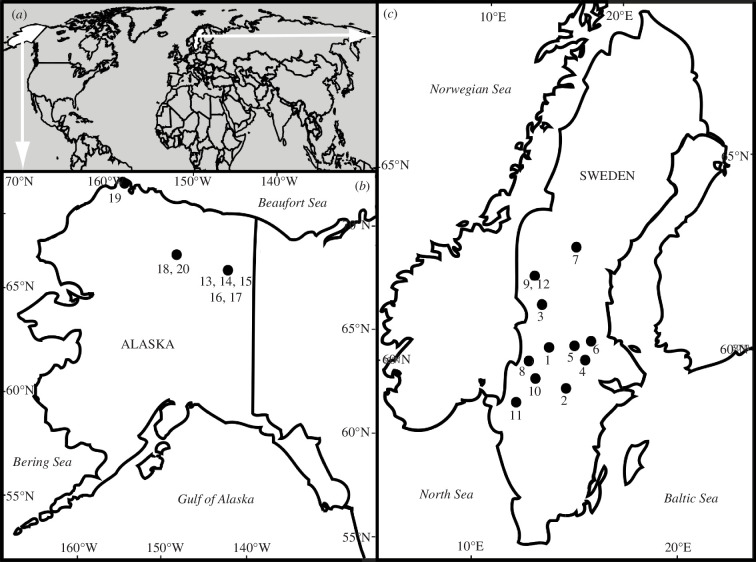
(*a*) Geographical overview with sampling localities of *C. lupus* in (*b*) Alaska and (*c*) Sweden, locality numbers referring to specimens given in electronic supplementary material, supplement 1.

**Figure 3.**
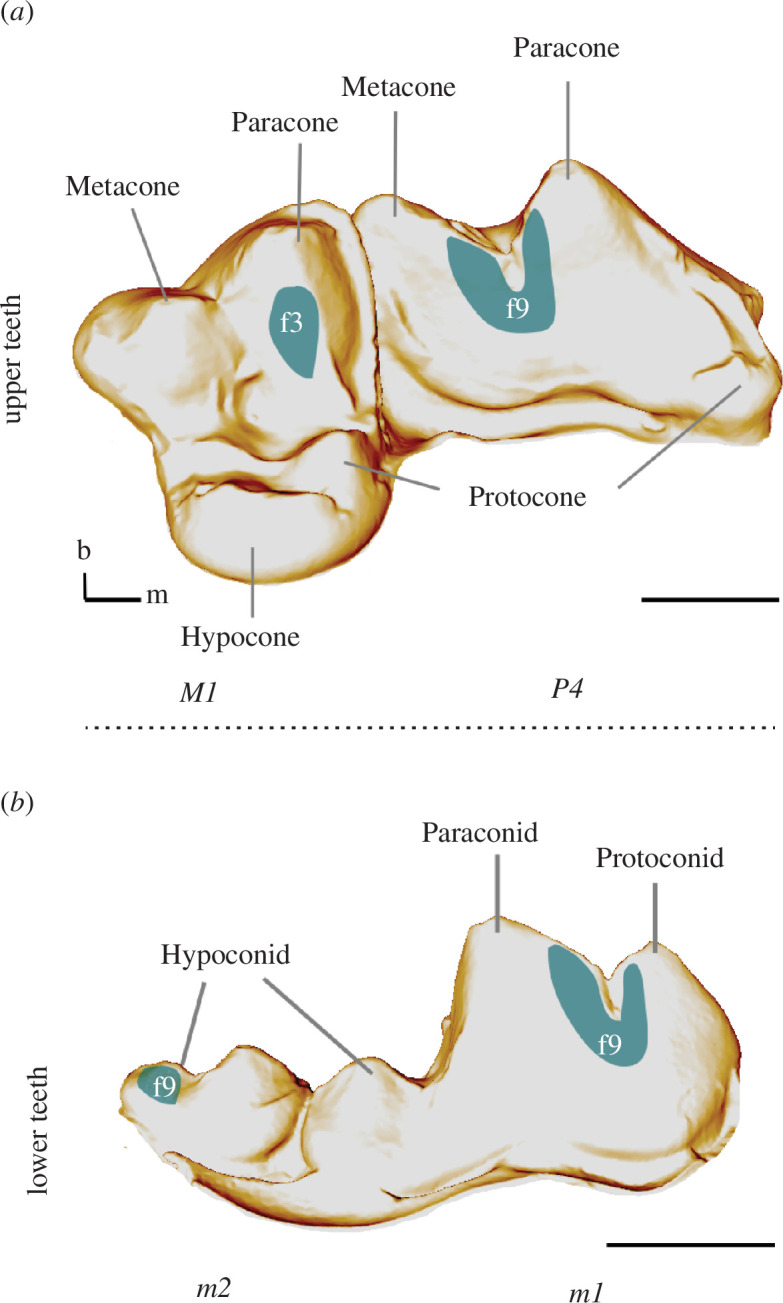
Morphology of the sampled tooth positions in *C. lupus* (NRM 20115027), (*a*) occlu-lingual view of upper fourth premolar (P4) and upper first molar (M1): facets 3 and 9 indicated, (*b*) buccal view of the sampled lower first m1 and lower second m2: facets 9 indicated (scale:1 cm; m: mesial, b: buccal).

The crania of 20 individuals of *C. lupus* Linnaeus, 1958 (12 specimens from Sweden, eight from Alaska) used for this study were curated at the Leibniz Institute for the Analysis of Biodiversity Change, Museum of Nature (Hamburg, ZMH) and Swedish Museum of Natural History (Stockholm, NRM). The ZMH specimens originated from three localities in Alaska (USA) and were shot during hunting trips from 1952 to 1964 (figure 2*a*). Sexual dimorphism data (female or male gender of the specimens) were indicated in the museum catalogue and used for dietary comparison between females and males. The specimens from the NRM were either shot in the wild, killed in traffic accidents or found dead owing to natural causes during the years 2010 to 2012 (figure 2*b*). All specimens died between December and March in the winter season. The individuals chosen for sampling had well-preserved premolars, molars, carnassial teeth (P4, M1) and lower second molars (figure 3). Specimens younger than 2 years (juveniles), as well as older specimens (>11 years) were excluded from sampling.

### 2.1. Moulding and casting

After cleaning the teeth with acetone, mouldings of the upper and lower jaws were made using the high-resolution A-silicone (Provil® novo Light C.D.2 regular set; Type 3; Heraeus Kulzer), following the procedure according to Schulz *et al*. [36,37] and Calandra *et al*. [38]

Facets were sampled in four positions (figure 3):

— the paracone shearing facet 9 on the carnassial P4;
— the posterior-lingual surface of the paracone facet 3 on M1; and
— the facet 9 on the anterior-lingual surface of the hypoconid on m1 and m2.

### 2.2. Data acquisition and processing

The three-dimensional measurements of the surface texture were conducted using the high-resolution confocal disc-scanning surface measuring system μsurf Custom (NanoFocus AG, Oberhausen, Germany) in accordance with Schulz *et al*. [36]. Four non-overlapping scans of the facet were taken using a 100× long distance lens. The data obtained were analysed in accordance with the three-dimensional industrial area surface texture standards (ISO 25178-2 [36–39]).

Primary surfaces were calculated by applying μsoft analysis premium software v. 6.2 (NanoFocus AG, Oberhausen, Germany; a derivative of Mountains® Analysis software by Digital Surf, Besançon, France), in line with the method of Schulz *et al*. [36–39]. Subsequently, 30 three-dimensional surface texture parameters according to ISO 25178-2 were applied to the S-F surface (for parameter list see electronic supplementary material, supplement 2). The parameters are divided into five parameter groups, describing height (*Sp, Ssk, Sku, Sq, Sv, Sz, Sa*), functional (*Smr, Smc, Sxp, Vm, Vv, Vmp, Vmc, Vvc, Vvv*), spatial (*Str, Std, Std*), hybrid (*Sdq, Sdr*) and feature/segmentation characters of the respective surfaces (*Spd, Spc, S10z, S5p, S5v, Sda, Sha, Sdv, Shv*).

### 2.3. Statistics

The arithmetical mean and standard deviation were calculated for the 30 parameters as descriptive statistics in accordance with Calandra *et al*. [38]: for each hypothesis, the complete set of 30 parameters was used, the data were trimmed to 15% to compensate for the non-normality and for groupwise comparison three statistical tests were accomplished. The robust Welch–Yuen (heteroscedastic omnibus) test [40,41] was coupled with a heteroscedastic pair-wise comparison test (analogous to Dunnett’s T3 test [42]) to detect significant differences within the arithmetical means. Additionally, the heteroscedastic rank-based test according to Cliff [43] was applied to compensate for non-normality and heterogeneity of the variances and to test whether the significance after the rank transformation was stable. This combination of tests has been found to perform better than the *F*-test ANOVA and countervails if the data are not heterogeneous or in a normal distribution [44,45]. Statistical analysis was conducted using the program R version 3.0.1 [46]. The following packages and their dependencies were used: xlsxReadWrite version 0.5.1 [47], doBy version 4.5.6 [48], R.utils version 1.25.2 [49] and grDevices version 3.0.1 [50].

## 3. Results

### 3.1. Intraspecific dietary separation (hypothesis 1)

**Figure 4.**
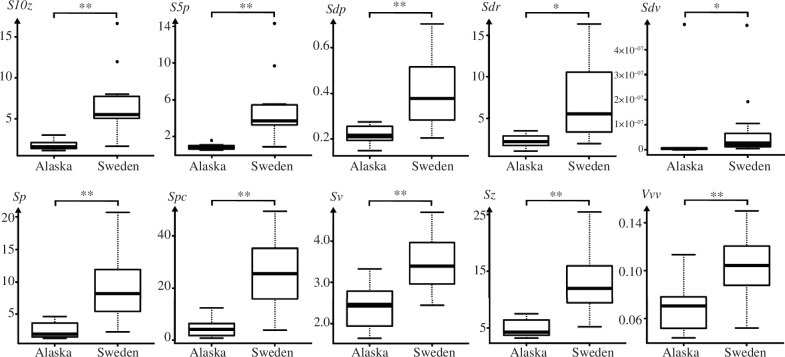
Texture parameters in two *C. lupus* populations (upper P4, facet 9). *S10z* is the 10-point height of the surface (pruning = 5%) (μm), *S5p* is the five-point peak height (pruning = 5%) (μm^3^ μm^−2^), *Sdq* is the root mean square gradient of the scale limited surface (%), *Sdr* is the developed interfacial area ratio of the scale limited surface (μm), *Sdv* is the mean closed dales volume (pruning = 5%) (μm^3 ^μm^−2^), *Sp* is the maximum peak height (μm), *Spc* is the arithmetic mean peak curvature (pruning = 5%) (μm), *Sv* is the maximum pit height (μm), *Sz* is the maximum height of the scale limited surface (μm^2^) and *Vvv* is the void volume of the valley (*p* = 80%) (no unit); asterisks (*) = levels of significance: *p*-value ≤ 0.05 (*), ≤0.01 (**), ≤0.01 (***).

**Figure 5.**
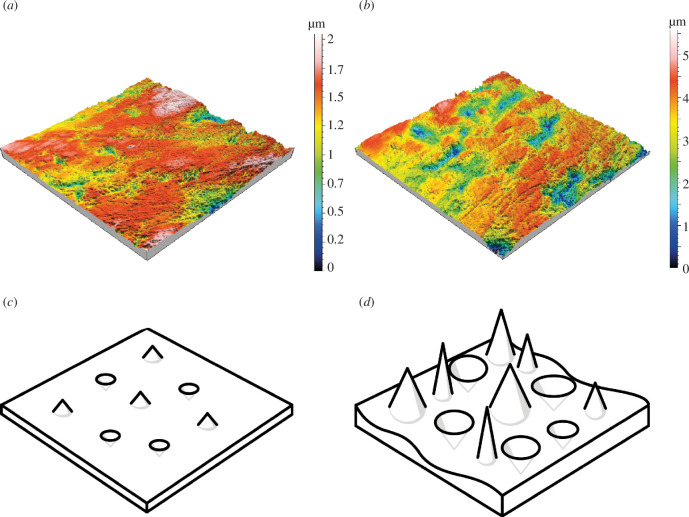
Representative meshed axiomatic three-dimensional models (160 × 160 μm) of enamel surfaces (upper P4, facet 9) of specimens from (*a*) Alaska, (*b*) Sweden. To illustrate differences in dale and hill features of the surface texture are translated into schematic models in line drawing of specimens from (*c*) Alaska and (*d*) Sweden.

In 10 out of the 30 parameters, the facets of Swedish wolves showed significantly higher values on facet 9 of the upper P4 than the facets of wolves from Alaska (figure 4, table 2). Swedish wolves featured higher peaks and deeper dales (figure 5; higher values of *Sp_p_*_< 0.006_, *Sv _p_*_= 0.004_ and *Sz_p_*_< 0.002_), displaying a rougher surface (higher values of *S10z_p_*_< 0.001_, *S5p _p_*_= 0.004_, *Sdq_p_*_< 0.001_, *Sdr_p_*_< 0.001_ and *Spc_p_*_≤ 0.001_) with more voluminous peaks and valleys (higher values of *Sdv_p_*_< 0.001_ and *Vvv_p_*_< 0.001_).

**Table 2.** Statistics for groupwise comparisons from Welch–Yuen, Dunnett's T3 and Cliff's tests (*p* values ≤ 0.05 in italics).

group	parameter	Welch–Yuen test				Dunnett’s T3 test			Cliff’s test			
		*Ft*	*p*	nu1	nu2	*Ft*	*Df*	*p*	*ph*	*pl*	*pu*	*p*
population: Sweden (*n*=8) *vs*. Alaska (*n*=12); facet 9, upper P4	*S10z*	19.8	*≤0.001*	1	70	4.5	70	*≤0.001*	0.73	0.60	0.83	*0.001*
	S5p	28.3	*≤0.001*	1	70	5.3	*70*	*≤0.001*	0.84	0.71	0.91	*≤0.001*
	*S5v*	17.0	*≤0.001*	1	49	4.1	49	*≤0.001*	0.82	0.70	0.90	*≤0.001*
	*Sda*	6.4	*0.014*	1	65	2.5	65	*0.014*	0.71	0.57	0.82	*0.004*
	*Sdq*	38.6	*≤0.001*	1	71	6.2	71	*≤0.001*	0.84	0.73	0.91	*≤0.001*
	*Sdr*	13.4	*≤0.001*	1	69	3.7	69	*≤0.001*	0.68	0.55	0.79	*0.009*
	*Sha*	15.8	*≤0.001*	1	71	4.0	71	*≤0.001*	0.73	0.60	0.83	*0.001*
	*Shv*	5.1	*0.028*	1	66	2.2	66	*0.028*	0.72	0.58	0.82	*0.003*
	*Sku*	4.0	*0.049*	1	58	2.0	58	*0.049*	0.65	0.51	0.77	*0.040*
	*Sp*	5.3	*0.025*	1	70	2.3	70	*0.025*	0.62	0.49	0.74	0.070
	*Sq*	9.4	*0.003*	1	67	3.1	67	*0.003*	0.70	0.57	0.81	*0.004*
	*Str*	4.8	*0.034*	1	53	2.2	53	*0.034*	0.65	0.51	0.77	*0.039*
	*Sv*	13.2	*0.001*	1	70	3.6	70	*0.001*	0.76	0.64	0.86	*≤0.001*
	*Sxp*	16.2	*≤0.001*	1	57	4.0	57	*≤0.001*	0.78	0.65	0.87	*≤0.001*
	*Vmc*	6.7	*0.012*	1	71	2.6	71	*0.012*	0.63	0.50	0.74	0.059
	*Vvv*	17.2	*≤0.001*	1	57	4.1	57	*≤0.001*	0.81	0.67	0.89	*≤0.001*
sex: female (*n* = 6) versus male (*n* = 6); both facet 3 and 9	*Sda*	9.4	*0.005*	1	30		*30*	*0.005*	0.74	0.58	0.86	*0.005*
	*Shv*	4.8	*0.036*	1	30		*31*	*0.036*	0.68	0.51	*0.82*	*0.043*
tooth row: upper P4 (*n* = 20), facet 3 versus upper M1 (*n* = 17), facet 9	*Sa*	5.4	*0.042*	1	10							
	*Sal*	12.3	*0.009*	1	7							
	*Smc*	7.3	*0.02*	1	11							
	*Std*	*5.2*	*0.043*	1	12							
	*Vm*	11.9	*0.015*	1	6							
	*Vmc*	7.0	*0.022*	1	12							
	*Vmp*	*11.9*	*0.015*	1	6							
	*Vv*	*7.7*	*0.018*	1	11							
	*Vvc*	8.8	*0.013*	1	11							
tooth jaw: upper M1 (*n* = 17, facet 3) versus lower m2 (*n* = 9, facet 9)	*Sdq*	8.8	*0.011*	1	13							
	*Sdr*	*10.7*	*0.004*	1	18							
	*Sq*	4.9	*0.049*	1	11							
	*Sv*	11.6	*0.004*	1	15							
	*Sxp*	6.6	*0.039*	1	7							
	*Vvv*	7.0	*0.039*	1	6							

*Notes:* ISO 25178 parameters: *S10z* is the 10-point height of the surface (pruning = 5%) (µm), *S5p* is the five-point peak heigh (pruning = 5%) (µm^3 ^µm^−2^), *Sa* is the arithmetic mean height/mean surface roughness (µm), *Sal* is the auto-correlation length (*s* = 0.2) (µm)*, Sda* is the mean dale area (pruning = 5%) (μm^3 ^μm^−2^), *Sdq* is the root mean square gradient of the scale limited surface (%), *Sdr* is developed interfacial area ratio of the scale limited surface (µm), *Sdv* is the mean closed dales volume (pruning = 5%) (µm^3 ^µm^−2^), *Smc* is the inverse areal material ratio (*p* = 10%), *Spc* is the arithmetic mean peak curvature (pruning = 5%) (µm), *Sp* is the maximum peak height (µm), *Sq* is the standard deviation of the height distribution, or RMS surface roughness (µm), *Std* is the texture direction (°), *Sv* is the maximum pit height (µm), *Sxp* is the peak extreme height difference in height between *p*% and *q*% (*p* = 50%, *q* = 97.5%) (µm), *Sz* is the maximum height of the scale limited surface (µm^2^), *Vm =* material volume at a given material ratio (*p* = 10%) (µm^3^ µm^−2^)*, Vmc* is the material volume of the core at given material ratio (*p* = 10%, *q* = 80%) (µm^3^ µm^−2^*), Vmp* is the material volume of peaks (*p* = 10%) (µm^3 ^µm^−2^)*, Vvc* is the void volume of the core (*p* = 10%, *q* = 80%) (µm^3 ^μm^−2^)*, Vv* is the void volume at a given material ration *p* = 10% (µm^3^ µm^−2^), *Vvv* is the void volume of the valley (*p* = 80%) (no unit), *Shv* is the mean hill volume (pruning = 5%) (µm^3^ µm^−2^).

*Df*, degree of freedom; *Ft*, test statistics; nu1, first-degree of freedom; nu2, second-degree of freedom; *p*, significance level; *ph*, test statistics; *pl*, lower 95% confidence interval; *pu*, upper 95% confidence interval.

**Figure 6.**
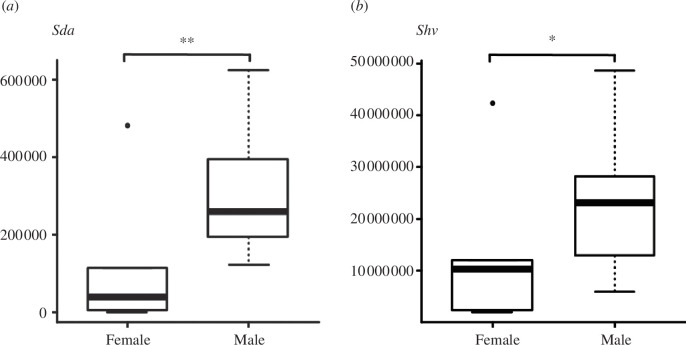
Boxplots of the texture parameters (*p* ≤ 0.05) of facet 9 on the upper fourth premolar of female (*n* = 6) and male (*n* = 6) Swedish wolves. (*a) Sda* = mean dale area (pruning = 5% μm^3^ μm^−2^), (*b*) *Shv* = mean hill volume (pruning = 5% μm^3^ μm^−2^); asterisks (*) = levels of significance: *p*-value ≤ 0.05 (*), ≤ 0.01 (**), ≤ 0.01 (***).

Male and female wolves from Sweden were tested to explore for sexual segregation reflected in the |three-dimensional surface textures (facets 3 and 9). In general, males were found to have larger dale area and hill volume (higher *Sda*_*p* ≤ 0.005_ and *Shv*_*p* ≤ 0.043_ values) when compared with female wolves (table 2, figure 6).

### 3.2. Tooth function (hypothesis 2)

**Figure 7.**
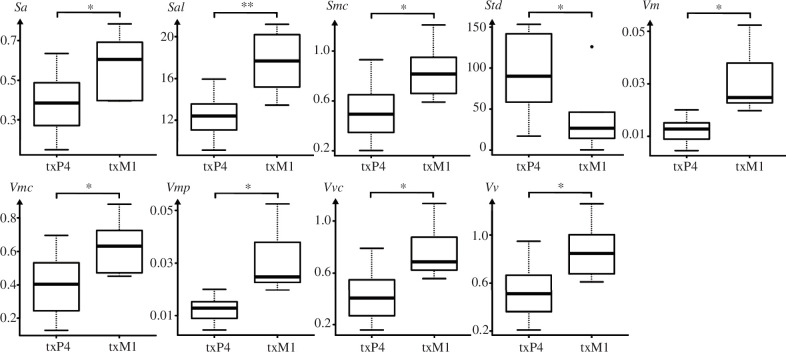
Texture parameters of the upper fourth premolar (P4) and the upper first molar (M1) in both populations, facets as indicated in table 2. *Sa* is the arithmetic mean height/mean surface roughness (μm), *Sal* is the auto-correlation length (*s* = 0.2; μm), *Smc* is the inverse areal material ratio (*p* = 10%), *Std* is the texture direction (°), *Vm* is the material volume at a given material ratio (*p* = 10%) (μm^3 ^μm^−2^), *Vmc* is the material volume of the core at given material ratio (*p* = 10%, *q* = 80%) (μm^3 ^μm^−2^), *Vmp* is the material volume of peaks (*p* = 10%) (μm^3^ μm^−2^), *Vvc* is the void volume of the core (*p* = 10%, *q* = 80%) (μm^3 ^μm^−2^), *Vv* is the void volume at a given material ration *p* = 10% (μm^3^ μm^−2^); asterisks (*) denote levels of significance: *p*-value ≤ 0.05 (*), ≤ 0.01 (**), ≤ 0.01***.

A comparison of the two upper tooth positions (upper P4, facet 9 versus upper M1, facet 3) revealed nine significant differences in texture signatures. Compared to the upper P4, the M1 was rougher (higher Sa_*p* ≤ 0.042_) and characterized by more voluminous peaks (higher values of *Sal*_*p* ≤ 0.009_, *Smc*_*p* ≤ 0.02_, *Vm*_*p* ≤ 0.015_, *Vmc*_*p* ≤ 0.022_, *Vmp*_*p* ≤ 0.015_, *Vvc*_*p* ≤ 0.013_, *Vv*_*p* ≤ 0.018_) (table 2, figure 7). The upper P4 had much higher values in texture direction (*Std* = 90°) when compared to the upper M1 (*Std* = 30°).

### 3.3. Antagonistic symmetry (hypothesis 3)

**Figure 8.**
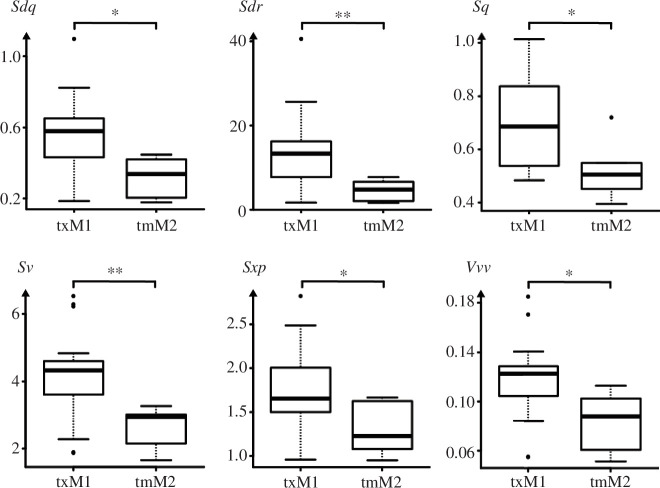
Texture parameter of the upper first molar (M1) and the lower second molar (m2). *Sdq* is the root mean square gradient (no unit), *Sdr* is the developed interfacial area ratio (%), *Sq* is the standard deviation of the height distribution, or RMS surface roughness (μm). *Sv* is the maximum valley height, depth between the mean plane and the deepest valley (μm), *Sxp* is the peak extreme height difference in height between *p*% and *q*% (*p* 1⁄4 50% and *q* 1⁄4 97.5%) (μm), *Vvv* is the void volume of the valleys at a given material ration *p* = 80% (μm^3^ μm^−2^); asterisks (*) denote levels of significance: *p*-value ≤ 0.05 (*), ≤0.01 (**), ≤0.01 (***).

A comparison of the occluding facet 3 of the upper first molar and the antagonistic lower second molar featured significant differences in six texture variables of the 30 parameters (table 2, figure 8). The upper M1 was rougher, and was characterized by larger and deeper dales with just a few peaks (higher *Sdq*_*p* ≤ 0.011_, *Sdr*_*p* ≤ 0.004_, *Sq*_*p* ≤ 0.048_, *Sv*_*p* ≤ 0.004_, *Sxp*_*p* ≤ 0.039_ and *Vvv*_*p* ≤ 0.039_ values).

## 4. Discussion

### 4.1. Population-specific dietary adaptation

Often studies using microwear and three-dimensional surface texture analyses focus on distinguishing the diet of different species [51–59]. In our study, we aimed to ascertain further subtle dietary signatures. Characterization of two geographically separate populations was expected to help resolve questions regarding intraspecific dietary adaptations. In Alaska as well as Sweden, wolves are known to be opportunistic feeders, consuming both meat and bones. Wolves from Alaska feed up to 51.5% on large prey such as moose. The other half of their diet consists of smaller prey mammals, birds and vegetation [12]. The average size of the Swedish wolves’ prey is 22.5% larger than that of the population from Alaska. We hypothesized that larger prey implied tooth contact with larger bones, which we proposed would in turn lead to comparatively more intense and possibly more frequent tooth–bone and tooth–cartilage contacts during the extraction of energy-rich tissues from the prey. Additionally, we expect less repetitive masticatory movements for smaller prey animals. This principle is also known to apply to durophagous fish [60] and indicates that the bigger the fish, the larger the prey animal that can be swallowed, though additional and probably also more powerful bites are needed to tackle a harder exoskeleton. Although our sample dealt with vertebrates having endoskeletons, our results were of a similar nature. We found higher peaks and deeper and larger valleys and hills as well as a rougher and more uneven surface texture on the enamel surface of the Swedish population, which consumed the larger prey. Smaller prey, as consumed by the population from Alaska, led to less rough surface textures. We were thus able to differentiate between the two geographically separated populations and test an ingesta-related hypothesis for prey consumption and availability. Our hypothesis predicting ingesta-specific texture patterns for both populations is thus supported by this dataset and confirms that surface textures reflect the size of prey as suggested also in specifically durophagous vertebrates [60,61]. Since the data relate to the posterior tooth positions of the wolves, where durophagous crushing action is performed, we also may consider the extent of durophagy as the major functional trait indicated by these data. We detected only minor sexual dimorphism in wolf skulls and dentitions (stature of canines, 3–8%) and corresponding to these morphological traits, we also expect three-dimensional surface textures to reflect minor sexual segregation, a hypothesis we consider supported by our data because male and female Swedish wolves are highly similar in their occlusal textures and only dale and valley parameters were sensitive to sex-specific differences. Such similarity may be the result of the shared hunting activities in the pack structure and equal access to resources for both alpha males and females [62]. However, we acknowledge that Swedish wolves are also highly inbred, which may have well contributed to this trait.

### 4.2. Tooth function (hypothesis 2)

Heterodonty is one of the key adaptations in mammals. The morphological differences in molar tooth positions may account for specific functional traits represented by antagonistic occlusal surfaces. We, therefore, tested if shear-cutting movements at the position of the carnassial (upper fourth premolar, P4) and the post carnassial upper first molar (M1) displayed distinct surface texture patterns. Our results showed that the upper first molar is rougher and characterized by more voluminous peaks and valleys (higher *Sal*, *Smc*, *Vm*, *Vmc*, *Vmp*, *Vvc*, *Vv*) in comparison to the upper premolar, which is part of the antagonistic carnassial system.

Spiky and voluminous peaks and valleys are indicative of durophagy but not of shear-cutting action and these textures are linked to a significant component of crushing activity performed between the post-carnassial molars. Pure shear-cutting activity, as performed by the upper fourth-premolar, results in much smoother surface textures. Our data are therefore further evidence for the functional difference between the carnassials and the post-carnassial molars in canids as demonstrated by Evans & Fortelius [35], Golliot *et al*. [8], Schubert *et al*. [9], Ungar *et al*. [10] and Severtsov *et al*. [34].

Carnivores exhibit predominantly orthal jaw movements, limiting lateral components to a minimum [35]. We found that the texture direction angle of 90° (*Std*) on the attrition-dominated facet of the upper P4 would immediately reflect those orthal movements. However, the lower angles of the texture direction (*Std* = 30°), as evident in the postcarnassial upper M1, should then be the result of abrasional ingesta, likely hard tissue contacts guided by the flanks of the tooth cusps. Those functional traits are further supported by larger valleys and fewer peaks and less rough texture on the lower second molar and support our hypothesis 2.

### 4.3. Antagonistic symmetry (hypothesis 3)

Lower cusp–ingesta contacts are proposed to always be less immediately prone to lateral peak loads as compared to upper cusp contacts during the prey processing phase. They rather act as stable antagonists with much less lateral components of movement, thereby increasing the probability that contacts remain in the same position in relation to the lower dentition while the upper contacts perform a lateral component of friction. We consider the rougher texture of the upper M1 as compared to the antagonistic m2 to immediately reflect this biomechanical constraint and thus consider hypothesis 3 supported by our data.

Subsequently, mastication will also likely affect antagonists differentially because lower cusps would experience some additional load by gravity, a mechanism so far only proposed by Kaiser & Fortelius [63] based on mesowear data of antagonistic equid molars. However, we acknowledge that the letter effect to our knowledge has never before been recognized in DMTA but should be minor as compared to the biomechanical constraints related to force introduction during early prey-processing activities.

We thus conclude that asymmetry observed in wear signatures of upper and lower molars, as more abrasion-dominated textural traits in upper cusps, should most likely be the result of biomechanical constraints of early prey-processing activities rather than just reflecting functional traits related to the mastication process in large carnivores. We can now understand the more pronounced asymmetry of upper and lower cusps we find in the Swedish population to reflect their reliance on large prey species (elk), demanding for prey processing oral activities transmitting significant lateral load at the ingesta–tooth functional surface interface.

Our data thus also support the evidence by Burtt & DeSantis [20] that diversity in dental functionality allows extant wolves to feed on a great diversity of prey animals and behave highly opportunistically in terms of their local dietary trait. DMTA can capture those traits and thus opens opportunities for including fossil predecessors of the wolf that may guide us to a better evolutionary understanding of the deep history of such dietary traits and the evolution of functional traits of their entire set of teeth.

## Data Availability

The datasets supporting this article have been uploaded as part of the supplementary material [64]. Raw data of surface texture parameter values is available via Zenodo [65].
